# Stefin A Regulation of Cathepsin B Expression and Localization in Cancerous and Non-Cancerous Cells

**DOI:** 10.3390/ijms26199321

**Published:** 2025-09-24

**Authors:** Anastasiia O. Syrocheva, Ekaterina P. Kolesova, Alessandro Parodi, Andrey A. Zamyatnin

**Affiliations:** 1 Research Center for Translational Medicine, Sirius University of Science and Technology, 354340 Sochi, Russia; syrocheva.ao@talantiuspeh.ru (A.O.S.); kolesova.ep@talantiuspeh.ru (E.P.K.); parodi.a@talantiuspeh.ru (A.P.); 2 Faculty of Bioengineering and Bioinformatics, Lomonosov Moscow State University, 119234 Moscow, Russia; 3 Belozersky Institute of Physico-Chemical Biology, Lomonosov Moscow State University, 119992 Moscow, Russia; 4 Department of Biological Chemistry, Sechenov First Moscow State Medical University, 119991 Moscow, Russia

**Keywords:** Cathepsin B, Stefin A, renal cell carcinoma, protease regulation, subcellular localization, cancer biomarkers, therapeutic targets

## Abstract

Cathepsin B (CTSB), a lysosomal cysteine protease, plays pivotal roles in cellular homeostasis and pathology, including cancer progression. This study investigates the regulatory interplay between CTSB and Stefin A (STFA), an endogenous inhibitor of cysteine proteases, in renal and prostate cancer cells. Using plasmid-based overexpression and silencing systems, we demonstrated that overexpressing STFA significantly reduces CTSB activity and protein levels, while silencing STFA leads to elevated CTSB activity and expression in cancer cells but not in non-cancerous cells (embryonic kidney cells—Hek293T and endothelial cells—EA.hy926). Furthermore, STFA modulates the subcellular distribution of CTSB, with STFA overexpression reducing nuclear CTSB levels and silencing inducing cytoplasmic accumulation in cancer cells. Colocalization analysis confirms a direct interaction between STFA and CTSB, highlighting the spatial coordination necessary for effective protease inhibition. These findings underscore the critical role of the CTSB-STFA axis in maintaining proteolytic balance and suggest potential therapeutic strategies targeting this interaction in renal carcinoma and other cancers.

## 1. Introduction

The lysosomal cysteine protease Cathepsin B (CTSB) [1] covers a key role in healthy and disease conditions [2,3] by regulating proteostasis in important processes such as autophagy, apoptosis, and extracellular matrix (ECM) remodeling [3,4]. CTSB is synthesized as a preproenzyme in the endoplasmic reticulum (ER), processed into a 46 kDa proenzyme, and further cleaved into a 31 kDa mature form in late endosomes. It is processed in a double-chain enzyme (25 kDa heavy chain, 5 kDa light chain) during lysosomal maturation [5]. Its catalytic triad (Cys108, His278, Asp292) enables pH-dependent activity [6], functioning as an exopeptidase in acidic lysosomes and an endopeptidase in neutral settings [6,7]. CTSB supports pro-hormone activation [8,9], ECM degradation [10], and lysosomal biogenesis via the MTORC1-TFEB pathway [11]. In cancer, CTSB showed a dual role. While it retains its homeostatic functions, excessive expression of CTSB has been linked to increased tumor aggressiveness [12,13]. Additionally, changes in CTSB levels during therapy can serve as a metabolic marker, reflecting tumor response and alterations in the tumor microenvironment [14,15]. This occurs through the degradation of ECM components [12] like collagen IV and laminin, the activation of oncogenic pathways such as MMP-9 and NF-κB [3], and the promotion of immune evasion.

Renal cell carcinoma (RCC), the most common form of kidney cancer, showed notable upregulation of CTSB, which correlates with advanced tumor stages, metastasis, and poor prognosis [16]. Together with other proteases, mendelian randomization studies have further established a connection between elevated CTSB levels and susceptibility to papillary RCC, underscoring its potential as a therapeutic target and biomarker [17,18]. The activity of CTSB is regulated meticulously across different levels. At the transcriptional level [19], regulation occurs through gene amplification, alternative splicing, and hypoxia-inducible factor (HIF)-1α-driven expression in tumors [20]. Post-translationally, pH-dependent conformational changes in the occluding loop occur, being closed in lysosomes to facilitate exopeptidase activity and open in extracellular environments for endopeptidase function [21]. Despite this complex regulation, endogenous inhibitors such as Stefin A (STFA) finely tune CTSB activity [16]. STFA, a member of the cytosolic cystatin superfamily, inhibits papain-like cysteine proteases via a tripartite wedge mechanism. In cancers like laryngeal [22] and esophageal squamous cell carcinoma [23], STFA overexpression diminishes CTSB-mediated invasiveness and proliferation without affecting CTSB protein levels, indicating a specific suppression of activity. A significant gap in knowledge persists regarding whether STFA can also regulate CTSB expression alongside its activity. Previous investigations focused on STFA’s enzymatic inhibition, while in this work we hypothesized that STFA influences CTSB expression and localization in renal cell and prostate cancer cells. To confirm this hypothesis, we evaluated CTSB expression levels and its colocalization with STFA in different cells types, including renal cancer cells (769p) where CTSB was shown to defining pathological aggressiveness [3], as well as embryonic renal cells (Hek293T) and prostate cancer cells (Du145), representing comparable models of normal and cancerous conditions. To ascertain the specificity of the effect initially identified in Hek293T cells, we per-formed a comparative analysis using endothelial cells (EA.hy 926 line). This approach was designed to establish whether the phenomenon represents a general cellular characteristic or is restricted to non-malignant cells.

## 2. Results

### 2.1. Effect of STFA on CTSB mRNA and Protein Expression

To investigate whether STFA expression directly influences CTSB expression, we utilized plasmids specifically designed to overexpress and silence STFA (pSTFA and pShSTFA, respectively). These plasmids were successfully tested in our previous studies and demonstrated high effectiveness [16]. All experiments were performed 48 h after transfection. We first determined the effect of increased STFA expression on CTSB expression by transfecting cancer cells with the pSTFA plasmid, in comparison with Hek293T immortalized not-cancerous cell line derived from embryonal renal tissue and known for its high transfection efficiency.

**Figure 1:**
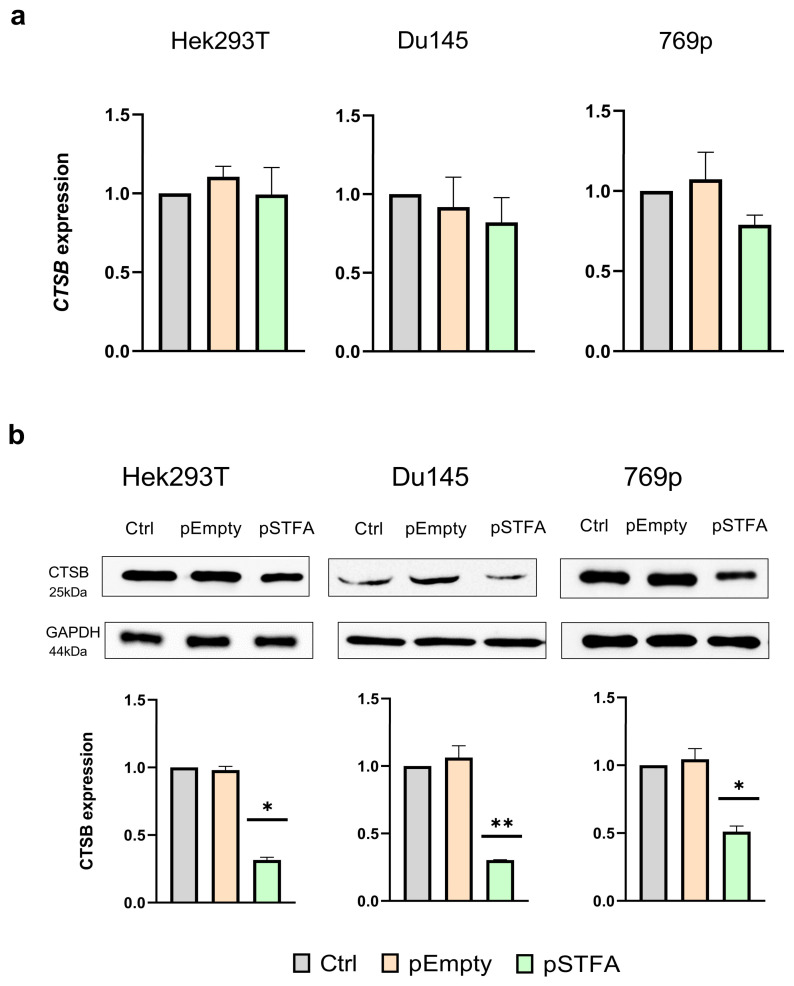
*CTSB* mRNA and protein expression following transfection with pEmpty and pSTFA plasmids: Cells were transfected with the different plasmids and mRNA (**a**) and protein (**b**) expression was evaluated after 48 h in comparison with untreated control. For each experiment, 3 biological and 3 technical replicates were performed. Results represent mean ± SD. * = *p* < 0.05, ** = *p* < 0.01.

Relative to untreated controls and empty plasmid (pEmpty) transfection, pSTFA induced a significant upregulation of *STFA* mRNA levels by 13-fold in Hek293T cells, 17-fold in Du145 cells, and over 100-fold in 769p cells (Figure S1a). Corresponding STFA protein levels increased 3-to 4-fold across all cell lines, although these changes did not fully mirrored the mRNA expression patterns. No significant alterations in STFA expression were detected following pEmpty transfection. Under the same experimental conditions, we also evaluated the impact of pSTFA on *CTSB* mRNA and protein expression. In all cell lines, transfection with pSTFA resulted in a slight decrease in *CTSB* mRNA expression compared to CTRL (Figure 1a) accompanied by a significant decrease in CTSB protein expression (Figure 1b).

Then, we transfected the cells with a plasmid targeting STFA expression (pShSTFA) and evaluated its effects on CTSB expression. We observed a significant reduction in *STFA* mRNA levels, with more than a 20-fold decrease in HeK293T and Du145 cells, and a 10-fold decrease in 769P cells accompanied by a significant decrease in protein expression in all the cell lines (Figure S2).

**Figure 2:**
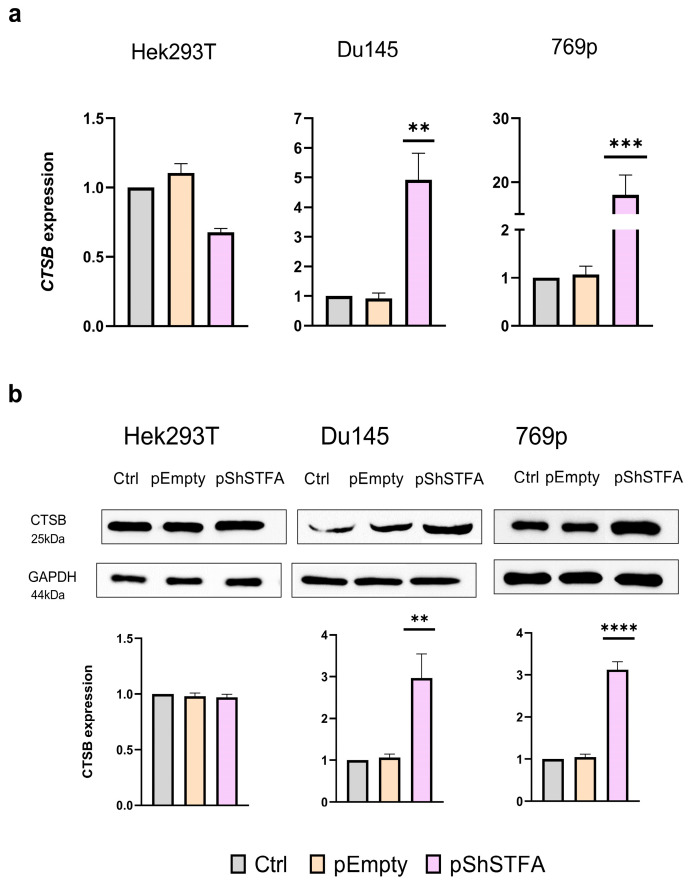
*CTSB* mRNA and protein expression following transfection with pEmpty and pShSTFA plasmids: Cells were transfected with the different plasmids and mRNA (**a**) and protein (**b**) expression was evaluated after 48 h in comparison with untreated control. For each experiment, 3 biological and 3 technical replicates were performed. Results represent mean ± SD. ** = *p* < 0.01, *** = *p* < 0.001, **** = *p* < 0.0001.

After confirming that our construct worked as intended, we measured the levels of *CTSB* mRNA and protein expression. In Hek293T cells, mRNA levels decreased in contrast to Du145 and 769p cells. Specifically, *CTSB* mRNA expression increased more than 4-fold in Du145 cells and over 15-fold in 769p cells (Figure 2a). Next, we assessed CTSB protein levels (Figure 2b). In line with the mRNA data, non-cancerous Hek293T cells exhibited a distinct behavior, as CTSB protein levels were comparable to the control. In contrast, both Du145 and 769p cancer cells showed a 3-fold increase in CTSB protein levels compared to the control.

### 2.2. CTSB Activity Is Dependent on STFA Expression

**Figure 3:**
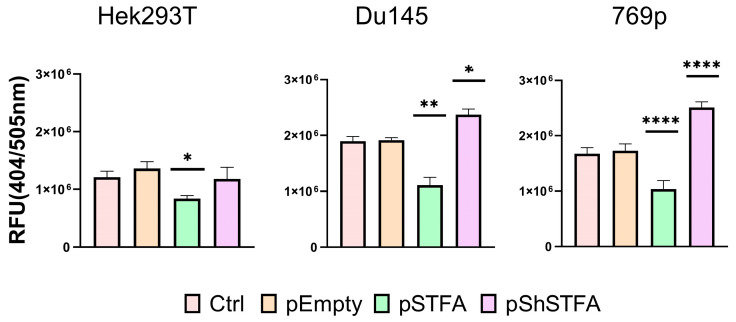
STFA assay for CTSB activity: CTSBB activity in human embryonic kidney Hek293T cells, human prostate cancer Du145 cells and human kidney cancer 769p cells after transfection with pEmpty, pSTFA and pShSTFA plasmids at 48 h. For each experiment, 3 biological and 3 technical replicates were performed. Results are presented as mean ± SD. * = *p* < 0.05, ** = *p* < 0.01, **** = *p* < 0.0001.

To further confirm our data, we investigated also CTSB activity (Figure 3). We detected that the basal activity of CTSB on Hek293T was lower than in the cancer cells studied. As anticipated, overexpression of STFA in all examined cell lines led to a reduction in CTSB activity. However, the effect of STFA silencing was ambiguous. In the embryonic cell line Hek293T, the activity level did not change compared to the control. In contrast, STFA silencing in the cancer cell lines resulted in a significant increase in CTSB activity.

In this effort, we also evaluated the effect of the target proteins on the viability of the cell lines. The cancer cell lines showed a tendency toward decreased viability with increased STFA levels, as well as with decreased CTSB levels; however, these changes were not statistically significant. In contrast to the cancer cell lines, embryonic Hek293T cells exhibited a statistically significant decrease in viability after transient transfection with pShSTFA, whereas the viability of the cancer cell lines increased under the same conditions (Figure S3).

### 2.3. CTSB Biodistribution Is Dependent on STFA Expression

**Figure 4:**
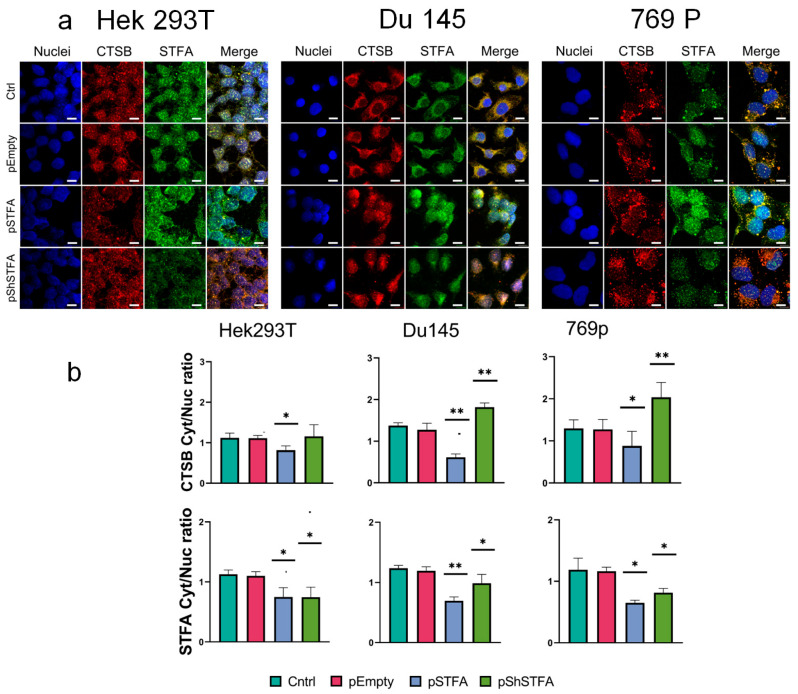
Effect of STFA expression on CTSB and STFA cytoplasmic and nuclear distribution: (**a**) Representative images showing the distribution of CTSB and STFA in human embryonic kidney cells-Hek293T, human prostate cancer cells-Du145, and human kidney cancer cells-769p. Nuclei were stained with blue (DAPI), CTSB was shown in red (Texas Red), and STFA in green (FITC channel). Scale bar: 10 μm. (**b**) Statistical analysis of the biodistribution of CTSB and STFA proteins in the nucleus and cytoplasm of the studied cell lines, presented as the ratio of cytoplasmic to nuclear signal intensity. All experiments were conducted 48 h after transfection with empty or STFA plasmids. For each experiment, 3 biological replicates were performed. Results are presented as mean ± SD. * = *p* < 0.05, ** = *p* < 0.01.

We then evaluated the cytoplasmic and nuclear distribution of CTSB and STFA (Figure 4) following transfection with both plasmids. We presented the data from this analysis as the ratio of signal intensities between these compartments. A consistent trend was observed across all cell lines studied. After transfection with pSTFA, the nuclear fraction of STFA predominantly increased, while the nuclear fraction of CTSB decreased. Silencing STFA with pShSTFA uniformly reduced STFA levels, resulting in data comparable to the control. However, the distribution of CTSB was disrupted: in cancer cell lines, its cytoplasmic fraction exceeded the nuclear fraction. In contrast, in the embryonic Hek293T cell line, the distribution of CTSB remained similar to the control, and its fluorescence intensity did not increase.

**Figure 5:**
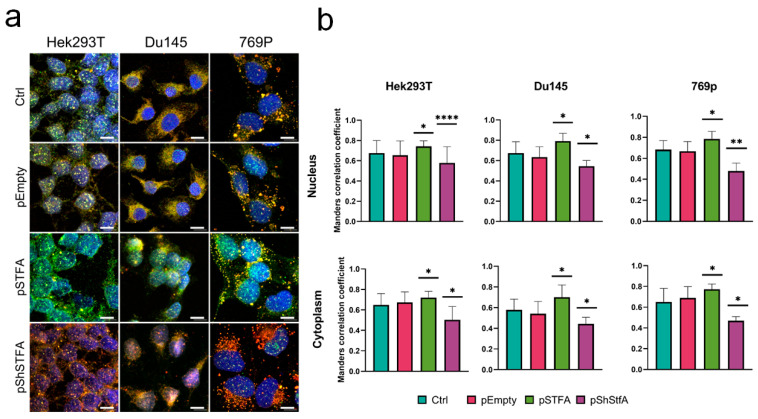
Colocalization analysis of CTSB and STFA: (**a**) Representative images showing the overlap of CTSB and STFA channels in human embryonic kidney cells (Hek293), human prostate cancer cells (Du145), and human kidney cancer cells (769p). Nuclei are stained in blue (DAPI), CTSB is shown in red (Texas Red), and STFA is shown in green (FITC channel). Scale bar: 10 µm. (**b**) Manders’ correlation coefficient analysis. For each experiment, 3 biological replicates were performed. Results are presented as mean ± SD. Statistical significance is indicated as follows: * = *p* < 0.05, ** = *p* < 0.01, **** = *p* < 0.0001.

Using the same set of images, we then quantified the colocalization of the two proteins in the nucleus and cytoplasm (Figure 5). For nuclear colocalization, all three cell lines exhibited similar behavior. The degree of colocalization increased when STFA levels were elevated (pSTFA) and decreased when STFA was silenced via transfection with pShSTFA. A similar pattern was observed for cytoplasmic colocalization.

**Figure 6:**
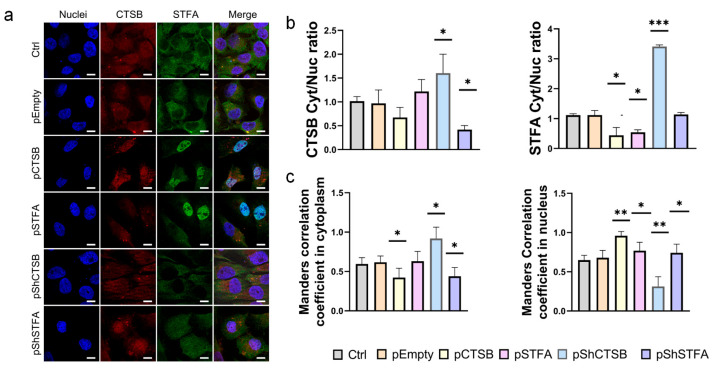
Effect of transfection on the cellular distribution of CTSB and STFA: (**a**) Representative images showing the distribution of CTSB and STFA in human endothelial cells EA. hy926. Nuclei were stained with blue (DAPI), CTSB was shown in red (Texas Red), and STFA in green (FITC channel). Scale bar: 10 μm. (**b**) Statistical analysis of the biodistribution of CTSB and STFA proteins in the nucleus and cytoplasm of the studied cell lines, presented as the ratio of cytoplasmic to nuclear signal intensity. (**c**) Manders’ correlation coefficient analysis All experiments were conducted 48 h after transfection with empty or STFA plasmids. For each experiment, 3 biological replicates were performed. Results are presented as mean ± SD. * = *p* < 0.05, ** = *p* < 0.01, *** = *p* < 0.001.

To grasp further the role of STFA expression in determining STFA and CTSB cellular distribution, we implemented these experiments with a human endothelial cell line (EA.hy926) as an additional control of non-cancerous cells. We transfected the cells with our plasmids for overexpression and silencing of the target genes (both CTSB and STFA) and assessed the subcellular distribution of CTSB and STFA in each case. In our previous study, transfection of Hek293T cells with a plasmid vector (pCTSB) carrying the cDNA (CTSB) did not reveal a corresponding increase in CTSB protein expression [24]. Similarly to Hek293T cells, we did not observe a reliable increase in CTSB levels following pCTSB transfection; however, STFA expression increased in both the pCTSB and pSTFA conditions. The level of STFA increased in the nucleus, as evidenced by microscopy analysis, while changes in CTSB levels were not statistically significant (Figure 6b). Correlation increased in the nucleus for both pCTSB and pSTFA transfections. After silencing CTSB via pShCTSB, we observed a decrease in the signal in both proteins, consistent with previous experiments. Transfection of endothelial cells with pShSTFA did not induce an increase in CTSB signal, a pattern also seen in HeK293T cells, confirming a similar regulation of STFA/CTSB axes in non-cancerous cells, apparently focus on maintaining cell homeostasis.

### 2.4. Effect of CTSB on STFA Expression and Distribution

To assess reversible effects in cancer and embryonic cell lines, we transfected cells with CTSB overexpression (pCTSB) and suppression (pShCTSB) plasmids and assessed levels of target proteins. Data presented in Figures S4 and S5 demonstrate that our plasmid constructs were functional, as evidenced by the successful modulation of CTSB expression. However, consistent with our recent report [24], we did not observe an increase in CTSB levels in Hek293T cells following transfection with the overexpression plasmid.

**Figure 7:**
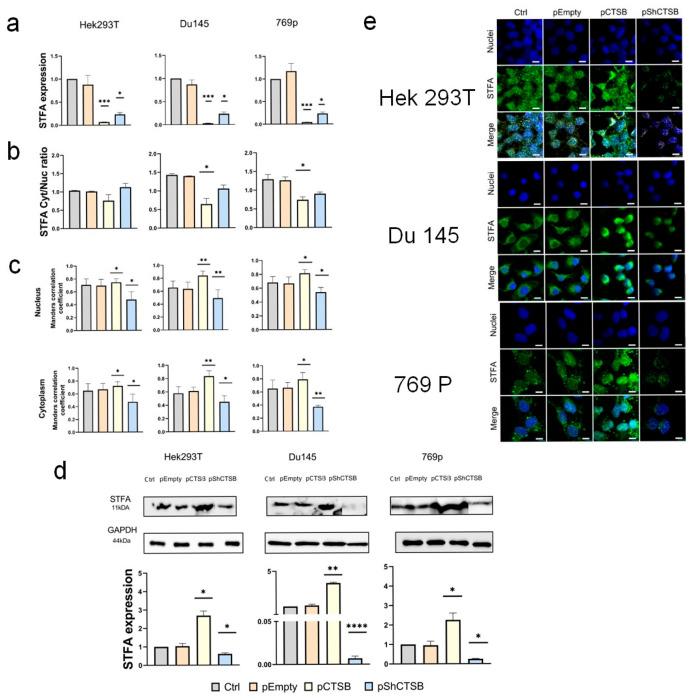
Effect of CTSB expression on STFA: (**a**) STFA mRNA level (**b**) Statistical analysis of the biodistribution of CTSB and STFA proteins in the nucleus and cytoplasm of the studied cell lines, presented as the ratio of cytoplasmic to nuclear signal intensity (**c**) Manders’ correlation coefficient analysis (**d**) STFA protein expression (**e**) Representative images showing the distribution of CTSB and STFA in human embryonic kidney cells—Hek293T, human prostate cancer cells — Du145, and human kidney cancer cells—769p. Nuclei were stained with blue (DAPI), CTSB was shown in red (Texas Red), and STFA in green (FITC channel). Scale bar: 10 μm All experiments were conducted 48 h after transfection with empty or STFA plasmids. For each experiment, 3 biological replicates were performed. Results are presented as mean ± SD. * = *p* < 0.05, ** = *p* < 0.01, *** = *p* < 0.001, **** = *p* < 0.0001.

The results regarding the effect of CTSB on STFA were inconclusive, as its overexpression caused a decrease in both *STFA* mRNA and protein levels. However, paradoxically, CTSB silencing also led to a reduction in these levels (Figure 7a,d). An analysis of the STFA cytoplasmic-to-nuclear (Cyt/Nuc) ratio showed that CTSB overexpression caused a reduction in this ratio across all cell lines, with the change being statistically significant in the cancerous lines. This pattern was consistent with the subcellular distribution of CTSB itself (Figure 7b,e; Figure S5). Taken together, these changes indicate a fine-tuned regulation of CTSB interactions in non-cancerous cell lines. Accordingly, CTSB silencing decreased Cyt/Nuc ratio for both proteins, indicating a predominant shift to the nuclear fraction. Finally, Manders’ correlation coefficient analysis showed an increase following CTSB overexpression, supporting the notion that STFA may form an inhibitory complex with CTSB to restore cellular balance, while the coefficient decreased upon CTSB knockdown, likely due to the overall reduction in the abundance of both target proteins (Figure 7c).

## 3. Discussion

In cancer, CTSB is implicated in processes such as extracellular matrix degradation [25], immune evasion [26], and activation of oncogenic pathways [27,28], contributing to tumor progression and metastasis [29]. Increased CTSB expression correlates with poor prognosis and increased aggressiveness [30]. Dysregulation of CTSB in cancer point out it potential role as a biomarker and therapeutic target. This study aims to investigate the regulatory relationship between CTSB and STFA, an endogenous inhibitor of cysteine proteases [31] in cancer cells.

Building on our previous investigation into the CTSB-STFA correlation in clinical samples and the 769p renal carcinoma cell line [16], the present study expands this work to a broader panel of models. Is this study, we employed an extended panel of cell lines to investigate mRNA expression, proteolytic activity, and cellular biodistribution. To ensure translational relevance and provide a non-cancerous control [32], we included the Hek293T human embryonic kidney cell line. The renal cell carcinoma line 769p was selected again for its high CTSB expression, a characteristic we previously documented [14]. Furthermore, mirroring the dependency observed in other cancer types, we included the prostate cancer line Du145 based on its well-documented reliance on CTSB activity for invasion [16]. While previous studies, have established a general inverse correlation between cystatins and cathepsin activity, the precise mechanistic interplay in renal and prostate cancer models remains less clear. Here, to directly interrogate this functional relationship, we employed plasmid-based overexpression and silencing of STFA. This approach allowed us to demonstrate, in a controlled manner, how STFA specifically modulates CTSB activity, protein stability, and sub-cellular distribution, thereby providing novel insights into the functional interplay within these specific oncogenic contexts.

We further confirmed the functionality of the plasmids by assessing their effect on STFA expression (Figures S1 and S2). Due to the limitations of plasmid-based approaches, temporal analyses were restricted, and a 48-hour time point was chosen for all experiments. This time point represents the peak of expression levels while minimizing the risk of plasmid loss. Previous studies highlighted CTSB’s role in cancer progression and STFA’s ability to inhibit this cysteine protease [21], focusing mostly on the role of STFA in reducing oncogenic potential [23], while under estimating its impact on CTSB expression or localization.

In all studied cell lines, STFA overexpression did not alter significantly *CTSB* mRNA levels, yet significant changes were observed at the protein level. Regardless of the tumorigenic potential of the cells, increased STFA expression consistently resulted in decreased active form of CTSB protein levels (Figure 1). This dissociation between mRNA and protein expression suggests a common mechanism of regulation of CTSB at the translational or post-translational levels [18] in response to increased STFA levels. In particular, we can speculate that the observed decrease in CTSB protein could be due to changes in its stability, linked to the interaction with its inhibitor STFA. Furthermore, this apparent reduction might not reflect a true decrease in CTSB synthesis or an increase in its degradation, but rather be an artifact caused by the formation of a stable, non-dissociable complex between CTSB and STFA. The nature of the bonds in the CTSB-STFA complex is primarily covalent. STFA is a tight-binding, irreversible inhibitor of cathepsin B. Once bound, a covalent bond is formed between a cysteine residue in the catalytic site of CTSB and a critical residue (often a carbonyl carbon) in the STFA molecule. This thioether bond is exceptionally strong and stable [33].

This regulatory relationship underscores a critical role for STFA in controlling CTSB also at the expression level, thereby influencing cellular proteolytic balance independently from transcriptional changes of the protease.

Following STFA silencing (Figure 2), an increase in CTSB expression was observed in cancer cell lines at both the mRNA and protein levels, whereas no significant change occurred in the non-cancerous Hek293T cells. The absence of altered *CTSB* mRNA expression in Hek293T cells may be attributed instead to transcriptional regulation mechanisms (i.e., higher stability [34] or transcriptional repression [35]). For instance, miR-214-3p has been shown experimentally to inhibit CTSB expression in AC16 cardiomyocyte cultures [35,36], while miR-140-5p has also been reported to regulate CTSB mRNA [37]. In contrast, cancer cell lines displayed a coordinated upregulation of CTSB transcript and protein levels following STFA knockdown, suggesting a disrupted regulatory environment where STFA downregulation relieves inhibition on CTSB expression. STFA inhibition may enhance CTSB expression via positive feedback. The absence of the inhibitor increases the availability of CTSB for various modifications, which stabilizes its mRNA and promotes increased protein levels. This mechanism enhances CTSB expression via both translational and post-transcriptional processes.

The comparative analysis in the noncancerous endothelial cell line EA. hy926 highlights the presence of an additional level of regulation of CTSB expression and activity. Following the upregulation of CTSB across all cell lines, we observed a concomitant decrease in *STFA* mRNA expression alongside an increase in STFA protein, suggesting an increased translational process or a post-translational regulatory mechanism. This modulation of translation efficiency could be potentially mediated by regulatory mechanisms including m6A RNA modification [38] and microRNAs [39] to normalize the CTSB-STFA balance. Conversely, CTSB silencing resulted in a reduction in both *STFA* mRNA and protein, corroborating our previous findings that CTSB activity is requisite for STFA production [24]. We therefore propose that this constitutes a natural homeostatic negative feedback loop, wherein STFA expression is fine-tuned post-translationally in response to CTSB levels.

These results suggest that, at least in non-cancerous cells, STFA expression serves as a universal mechanism for regulating CTSB levels, influencing both its proteolytic activity and overall expression.

Upon increasing STFA expression across all cell lines studied, we observed a consistent reduction in CTSB activity (Figure 3), in agreement with previous reports demonstrating STFA’s inhibitory effect on cysteine proteases such as CTSB [16]. However, the effects of STFA silencing were less straightforward. Notably, only in the non-cancerous Hek293T cells STFA knockdown did not result in increased CTSB activity, a finding that is consistent with the multi-level regulation of cysteine proteases by the cystatin superfamily. Previous studies have shown that multiple cystatins can compensate for each other in the regulation of cathepsin activity [40,41]. This compensatory mechanism of competitive inhibition may explain why the reduction in STFA alone was insufficient to increase CTSB activity in Hek293T cells, as non-cancerous cells often maintain a robust regulatory network to preserve cellular homeostasis. In contrast, cancer cell lines, which frequently exhibit dysregulation of protease inhibitors, displayed significant increases in CTSB activity following STFA knockdown. This phenomenon is consistent with current literature suggesting that loss of cystatin function can exacerbate protease-mediated activity, like in the case of extracellular matrix degradation and cell death pathways’ amplification [25,42]. Our data highlight the key role of STFA-CTSB correlation in the maintenance of proteolytic balance, especially in cells with low oncogenic potential.

The subcellular localization of CTSB is critically important for cellular function. An increase in nuclear CTSB levels can have important consequences, on cell biology. Nuclear CTSB localization was associated with proper chromosome segregation during mitosis [43] and DNA damage during oncological transformation [44]. Previous studies have reported nuclear localization of both CTSB and STFA, a finding that we corroborated in our experiments [16]. Previous studies have shown that human renal cell carcinoma 769p cells produce over three times more CTSB than embryonic HeK293T cells. However, both cell types exhibit a similar ratio of CTSB distribution between the nucleus and cytoplasm [32], a finding confirmed in this study.

Moreover, here we demonstrated that STFA expression modulation affects the subcellular distribution of both proteins (Figure 4). Notably, STFA knockdown via pShSTFA induced a redistribution characterized by increased CTSB levels in both nuclear and cytoplasmic compartments. Interestingly, the reduction in STFA signal was uneven; the nuclear fraction of STFA remained predominant relative to the cytoplasmic pool. This suggests a possible protective mechanism whereby retained nuclear STFA might mitigate the harmful effects associated with elevated nuclear CTSB.

Previous research on CTSB and STFA has largely focused on their expression and functional interplay, but investigations into their colocalization remain limited despite its critical relevance to confirming direct molecular interactions. Our study demonstrates that the degree of colocalization between CTSB and STFA correlates with STFA expression levels, supporting the hypothesis of a spatially coordinated inhibition (Figure 5). Colocalization analysis revealed that increased STFA levels enhance CTSB-STFA overlap in both nuclear and cytoplasmic compartments, facilitating effective protease regulation. Conversely, silencing STFA disrupts this spatial coordination, leading to aberrant CTSB distribution and activity in cancer cells. These findings emphasize the biological significance of colocalization in maintaining proteolytic balance and cellular homeostasis, particularly in pathological conditions such as RCC.

## 4. Materials and Methods

### 4.1. Cell Lines

Three human cell lines with varying basal levels of CTSB, as indicated by the GEPIA (Gene Expression Profiling Interactive Analysis) database, were included in this study. The prostate cancer cell line Du145 exhibits lower CTSB expression compared to healthy prostate cells, while the kidney cancer cell line 769p is characterized by increased CTSB levels compared to normal tissue. Hek293T cells, derived from human embryonic kidney, were used as a healthy control. Human endothelial cells EA.hy926, a hybrid of HUVEC umbilical vein cells and A549 lung cancer cells, were also used as a healthy control. The cell lines derived from human were purchased from American Type Culture Collection. Cells were grown in the recommended medium (DMEM or RPMI) supplemented with 10% fetal bovine serum and 1% penicillin-streptomycin antibiotic mixture (all from Gibco, Waltham, MA, USA) at 5% CO_2_ and 37 °C in a humidified atmosphere containing 5% CO_2_. Cell lines were checked with the MycoReport (Evrogen, Moscow, Russia) and were free of contamination.

### 4.2. Transfection

Cells were grown to 70% confluence and transfected with pcDNA3.1-STFA. The plasmid with the STFA sequence was kindly provided by Dr. Yuan Chen (Universitätsklinikum J07747, Jena, Germany). The CTSB overexpression plasmid contained the human CTSB sequence and was described previously. The gene expression silencing plasmid (pShSTFA or pShCTSB) contained short hairpin RNA (shRNA) and was described in detail previously [16].

### 4.3. Cathepsin B Activity Assay

48 h after transfection, cells were lysed and activity was assessed using the Cathepsin B Fluorometric Activity Assay (ab65300) (Abcam, Cambridgeshire, UK). Cell lysis and sample preparation were performed according to the manufacturer’s protocol. Fluorescence was measured using a CLARIOstar^®^ Plus reader (BMG labtech, Ortenberg, Germany).

### 4.4. RNA Isolation and Real-Time Polymerase Chain Reaction (RT-qPCR)

Total RNA was extracted from the cells using the PureLink™ RNA Kit (Invitrogen, Waltham, MA, USA) according to the manufacturer’s instructions. Complementary DNA (cDNA) was synthesized from mRNA using the MMLV cDNA synthesis kit (Evrogen, Moscow, Russia). For reverse transcription, 1 µg of total RNA with an OD260/OD280 ratio of 1.7–2.0 (measured by NanoDrop One, ThermoFisher, Waltham, MA, USA) was used. Gene expression levels were quantified by RT-qPCR using cDNA as a template in reactions containing 5X qPCRmix-HS (Evrogen, Moscow, Russia) and specific primers: CTSB (F-5′-TTCTTGCGACTCTTGGGACTTC-3′, R-5′-TGACGAGGATGACAGGGAACTA-3′), STFA (F-5′-TCTTCCCGGACAAAATGAGG-3′, R-5′-GACTCAGTAGCCAGTTGAAGG-3′), and GAPDH (F-5′-CTTCGCTCTCTGChTCCTCCTGTTCG-3′, R-5′-ACCAGGCGCCCAATACGACCAAAT-3′). PCR reactions were performed in triplicate under the following conditions: 95 °C for 30 s, followed by 40 cycles of 95 °C for 5 s, 60 °C for 15 s, and 72 °C for 10 s, using the StepOne Real-Time PCR System (Applied Biosystems, Waltham, MA, USA). Gene expression levels were analyzed using the ddCT method.

### 4.5. Western Blotting

Following transfection, cells were washed twice with ice-cold PBS, harvested, and resuspended in lysis buffer containing 50 mM Tris-HCl (pH 8.0), 100 mM NaCl, 0.5% NP-40, 1% Triton X-100, and 1 × protease inhibitor cocktail (ThermoFisher, Waltham, MA, USA). Protein concentrations in the cell lysates were quantified, and 50 μg samples were separated on 12% Tris-glycine gels for CTSB and 16% Tris-glycine gels, then transferred to PVDF membranes (Bio-Rad, Hercules, CA, USA).

CTSB and STFA expression was detected using specific rabbit polyclonal primary antibodies for CTSB and STFA (Cloud-Clone Corp., Wuhan, China). After extensive washing, the membranes were incubated with goat anti-rabbit IgG (HRP) secondary antibody (Abcam, Cambridge, UK) in 5% non-fat milk in PBST. Reactive bands were visualized using enhanced chemiluminescence (Bio-Rad, USA). GAPDH was used as a loading control, detected with rabbit polyclonal anti-GAPDH primary antibodies (Cloud-Clone Corp., Wuhan, China) and goat anti-rabbit IgG (HRP) secondary antibody (Abcam, Cambridge, UK).

### 4.6. Immunofluorescent Staining

The cells were seeded on glass slides, grown to 70% confluency, transfected, and fixed after 48 h. For this purpose, cells were washed with D-PBS, fixed with 1% PFA/PBS for 15 min, and permeabilized with 0.25% Triton^®^ X-100 for 10 min. After blocking non-specific binding sites with 2% BSA/PBS-T, cells were incubated for 4 h with primary antibodies: mouse monoclonal anti-CTSB (Abcam, Cambridge, UK) and rabbit polyclonal anti-STFA (Cloud-Clone Corp., Wuhan, China). Following this, cells were incubated for 1 h at room temperature with fluorophore-labeled secondary antibodies. CTSB was detected using a TexasRed-labeled secondary antibody (FineTest Corp. Wuhan Fine Biotech Co., Ltd., Wuhan, China). STFA was detected using a FITC-labeled secondary antibody (Cloud-Clone Corp., Wuhan, China). Nuclei were counterstained with 0.5 µg/mL DAPI (Bio-Rad, Hercules, CA, USA). Confocal fluorescence images were acquired using an inverted point-scanning confocal microscope (LSM 980 Airyscan on an Axio Observer 7, Carl Zeiss Microscopy GmbH, Jena, Germany) equipped with a motorized piezo stage and both 20 × and 63 × oil immersion objective lenses (Carl Zeiss Microscopy GmbH, Jena, Germany). Airyscan images were processed using the Airyscan processing algorithm (strength 3.9) and captured with Zen software (version Zen Blue 3.2, Carl Zeiss Microscopy GmbH, Jena, Germany). The images were analyzed using the CellProfiler 4.2.4 program with settings that separated signals from the cytoplasm and nucleus. Images with a lens magnification of 20 and a zoom magnification of 2, 3 images of different areas from each slide, were used for the analysis.

### 4.7. Statistical Analysis

Statistical processing was carried out in GrafPad 9.5 for Windows, Graph Pad software San Diego, CA, USA. The results of relative expression analysis are presented as mean ± SD for normal distribution. The *p*-value < 0.05 was considered statistically significant with * *p* < 0.05, ** *p* < 0.01, and *** *p* < 0.001.

## 5. Conclusions

This study redefines the critical regulatory relationship between CTSB and its endogenous inhibitor STFA, demonstrating their interplay in cancer and non-cancerous cells. In cancer cells, STFA silencing leads to increased CTSB expression and activity. Conversely, non-cancerous cells exhibit robust compensatory mechanisms to maintain protease regulation, underscoring STFA’s role as a stabilizing factor in cellular homeostasis, highlighting this cross-talk as a potential molecular target. Moreover, the subcellular localization of CTSB and STFA plays a pivotal role in their functional interaction, with nuclear STFA potentially mitigating the harmful (pro-proliferative) effects of elevated nuclear CTSB. Colocalization analysis further revealed that STFA expression enhances spatial coordination with CTSB, ensuring effective protease regulation, while its silencing disrupts this balance in cancer cells. The majority of CTSB has been detected in the cytoplasm (likely in the endoplasmic reticulum for further maturation), while STFA is predominantly cytosolic.

This study highlights the critical role of STFA in preserving proteolytic balance and cellular stability, emphasizing its function as a key regulator of CTSB—and potentially other cysteine cathepsins—in cancerous cells, since its absence in non-cancerous cells was compensated by alternative regulatory mechanisms.

## Data Availability

Data are contained within the article and Supplementary Materials.

## Supplementary Materials

The following supporting information can be downloaded at: https://www.mdpi.com/article/10.3390/ijms26199321/s1.
