# Supplementary material for: Stefin A Regulation of Cathepsin B Expression and Localization in Cancerous and Non-Cancerous Cells
Source: Int J Mol Sci. 2025 Sep 24;26(19):9321. doi: 10.3390/ijms26199321 (PMC12524445; doi:10.3390/ijms26199321)
Supplement: Supplementary file 1 [file ijms-26-09321-s001.zip › ijms-3833866-supplementary.pptx]

## Slide 1
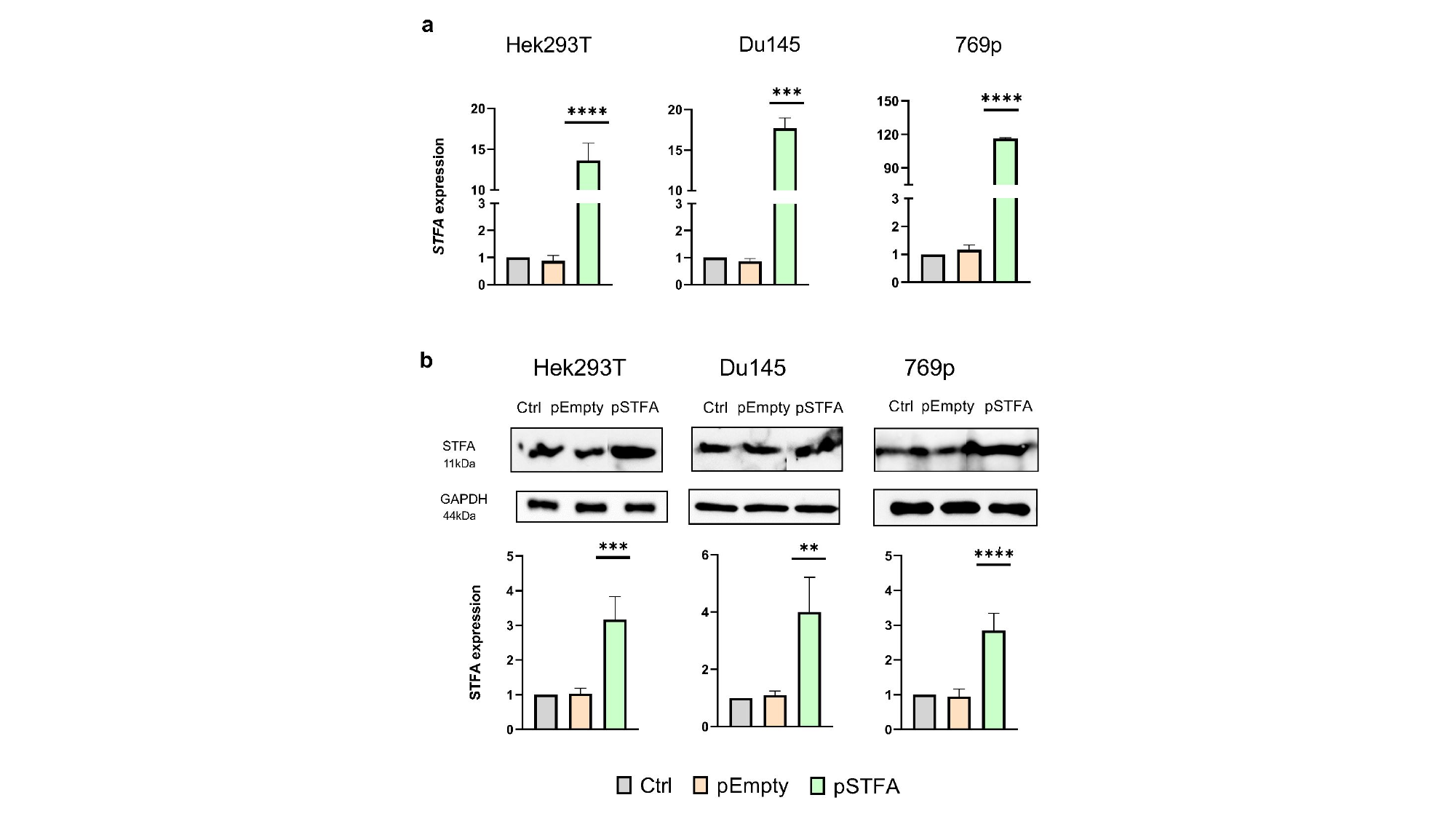

## Slide 2
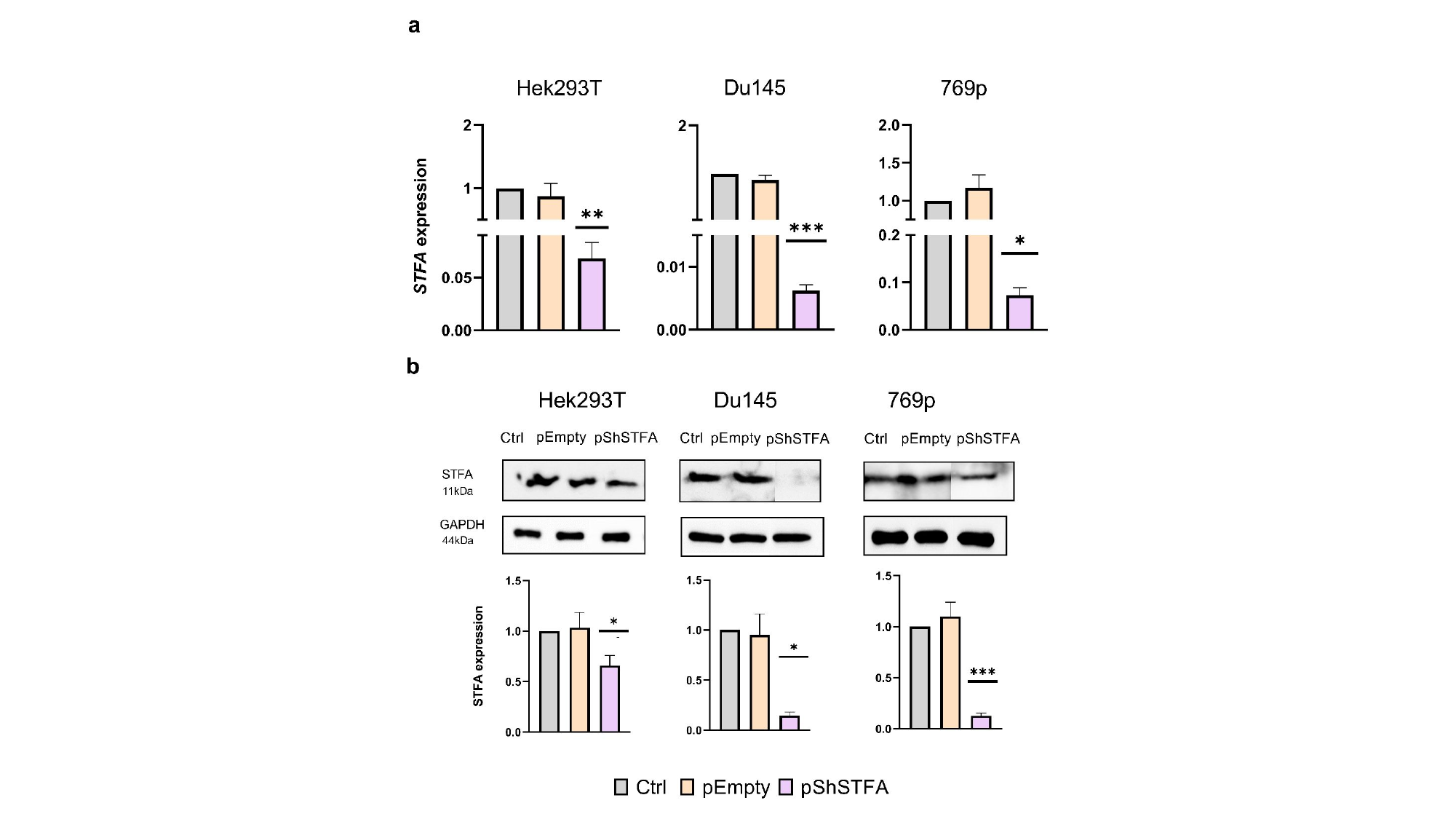

## Slide 3
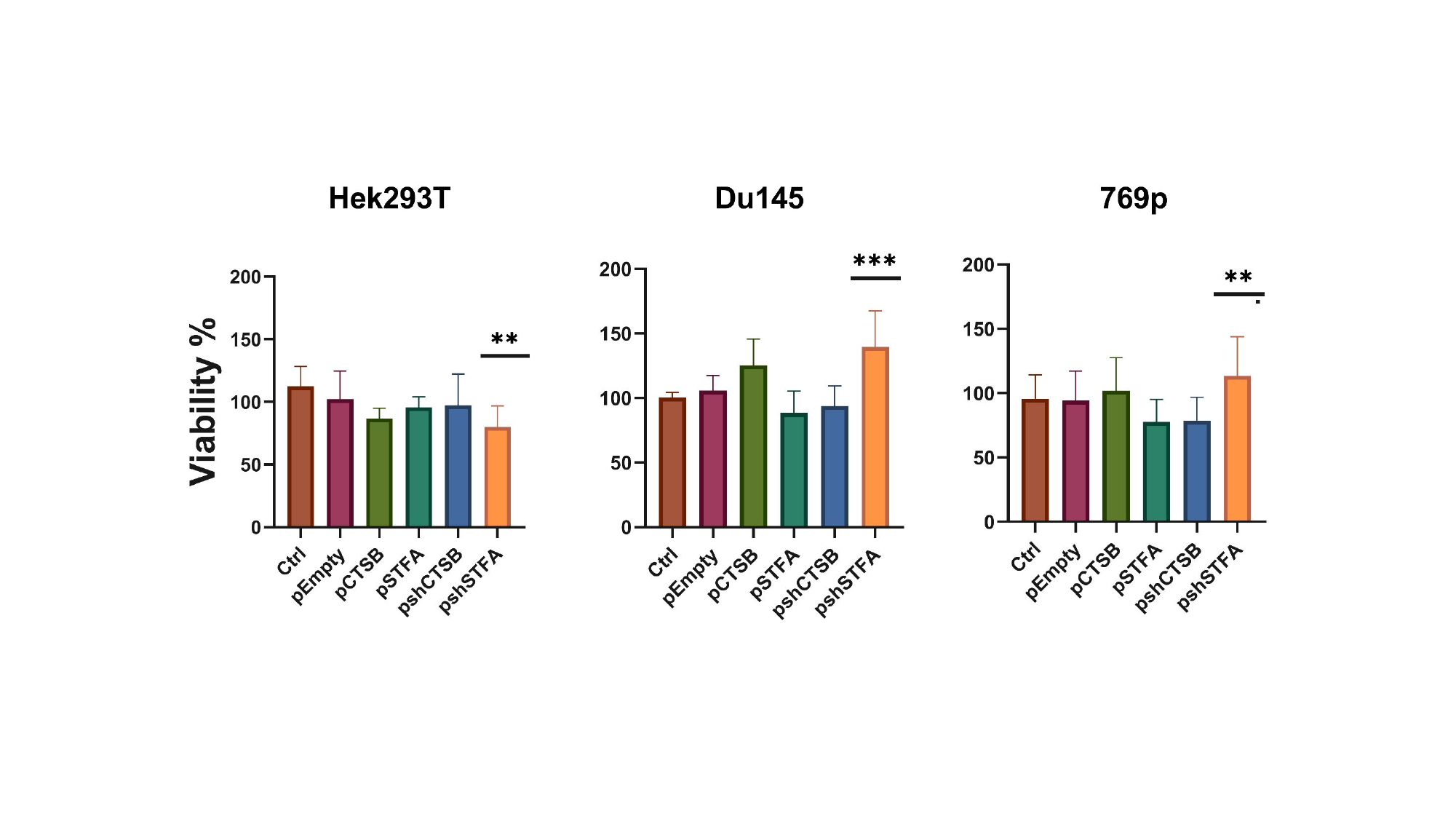

## Slide 4
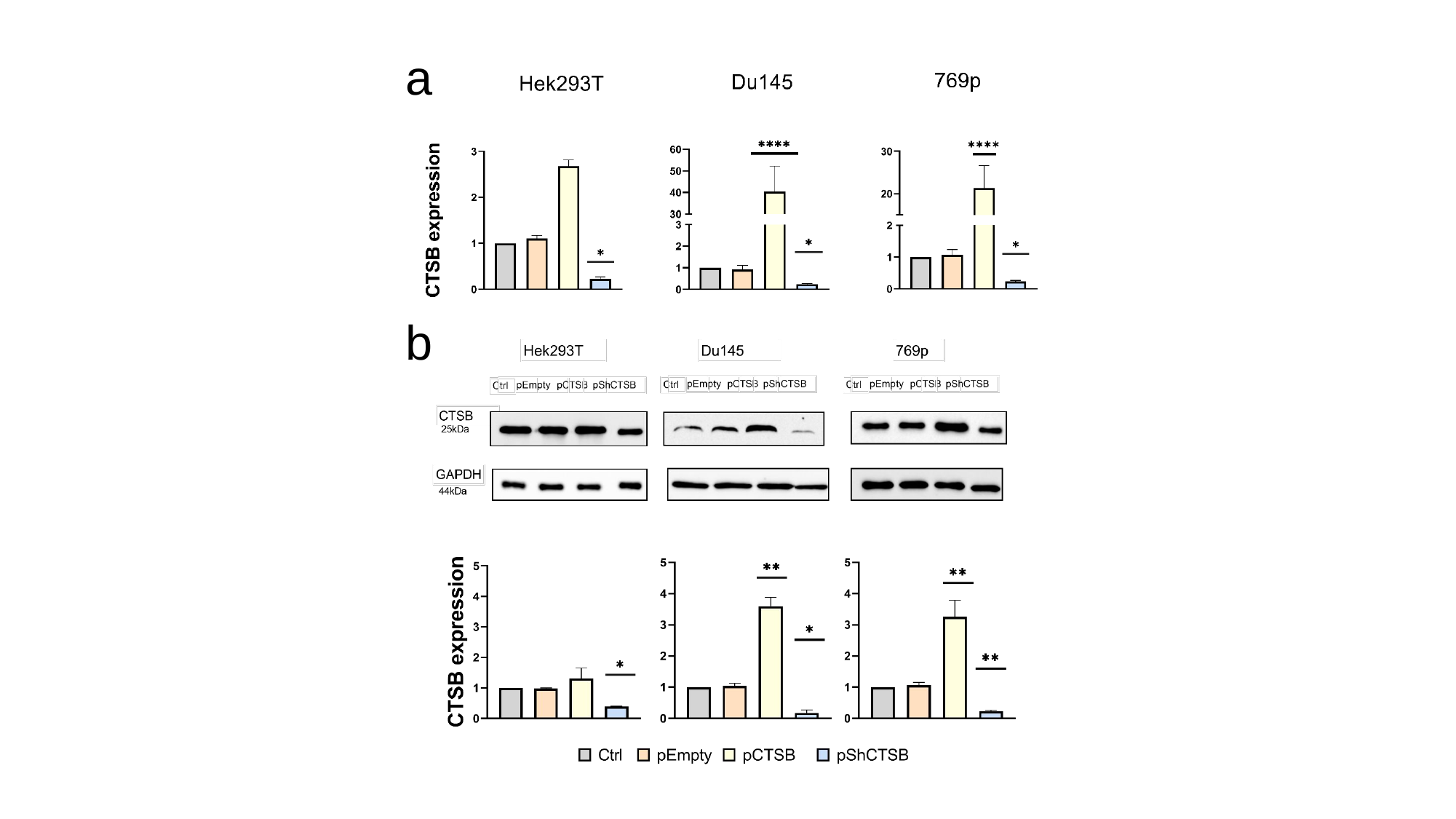

a
b

## Slide 5
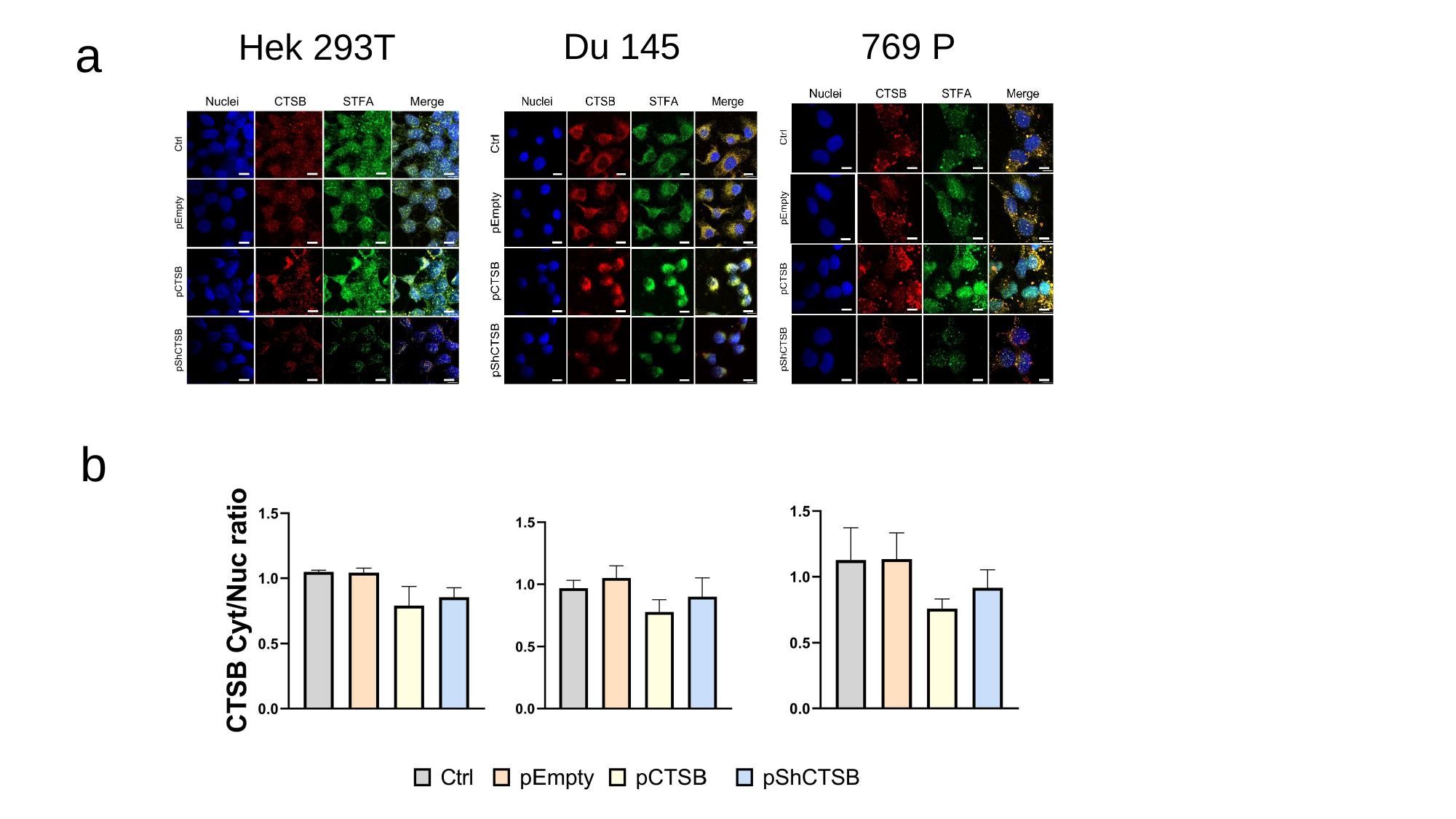

Du 145
769 P
a
Hek 293T
b

## Slide 6
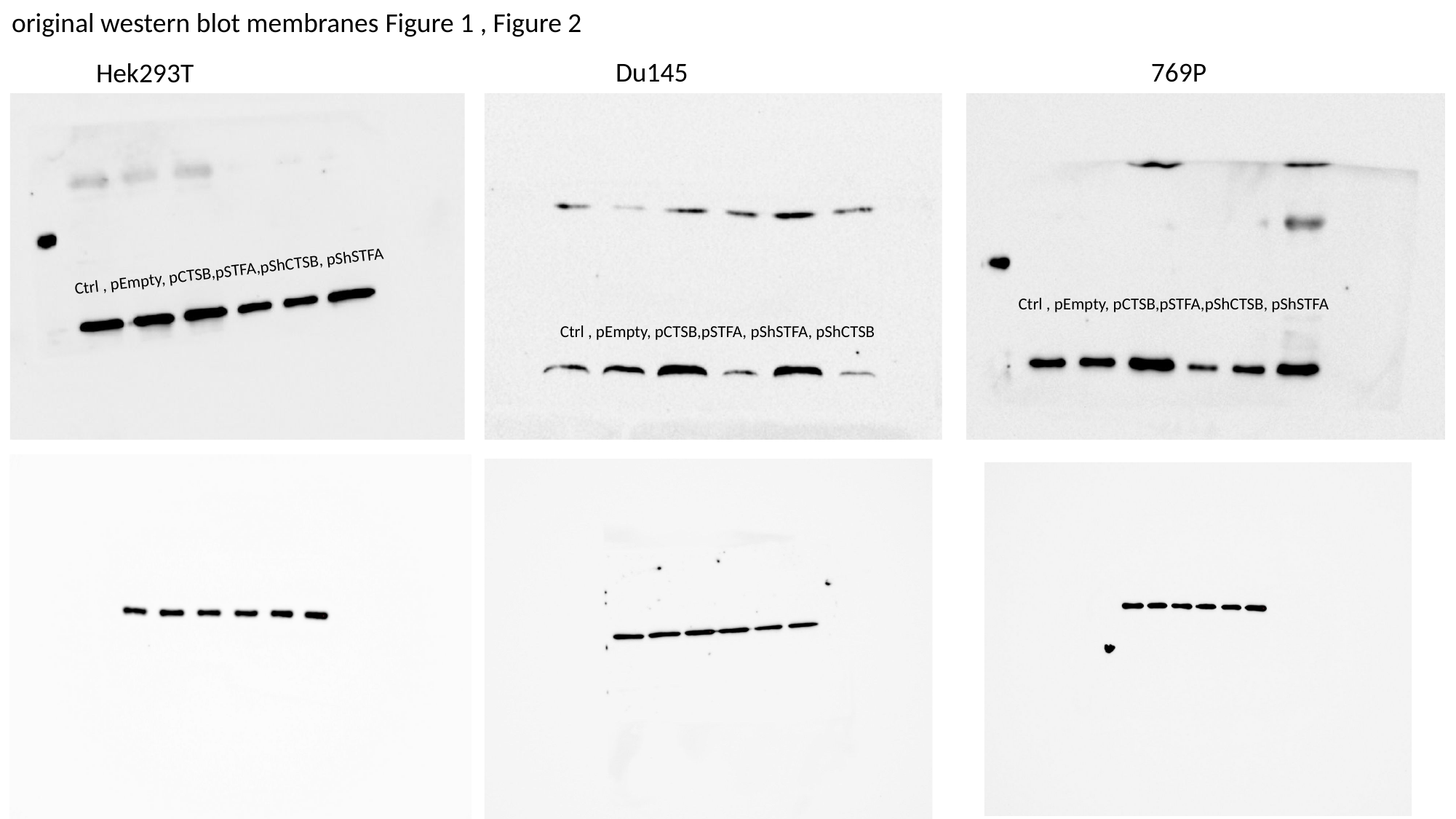

original western blot membranes Figure 1 , Figure 2
Du145
769P
Hek293T
Ctrl , pEmpty, pCTSB,pSTFA,pShCTSB, pShSTFA
Ctrl , pEmpty, pCTSB,pSTFA,pShCTSB, pShSTFA
Ctrl , pEmpty, pCTSB,pSTFA, pShSTFA, pShCTSB
Ctrl , pEmpty, pCTSB,pSTFA,pShCTSB, pShSTFA

## Slide 7
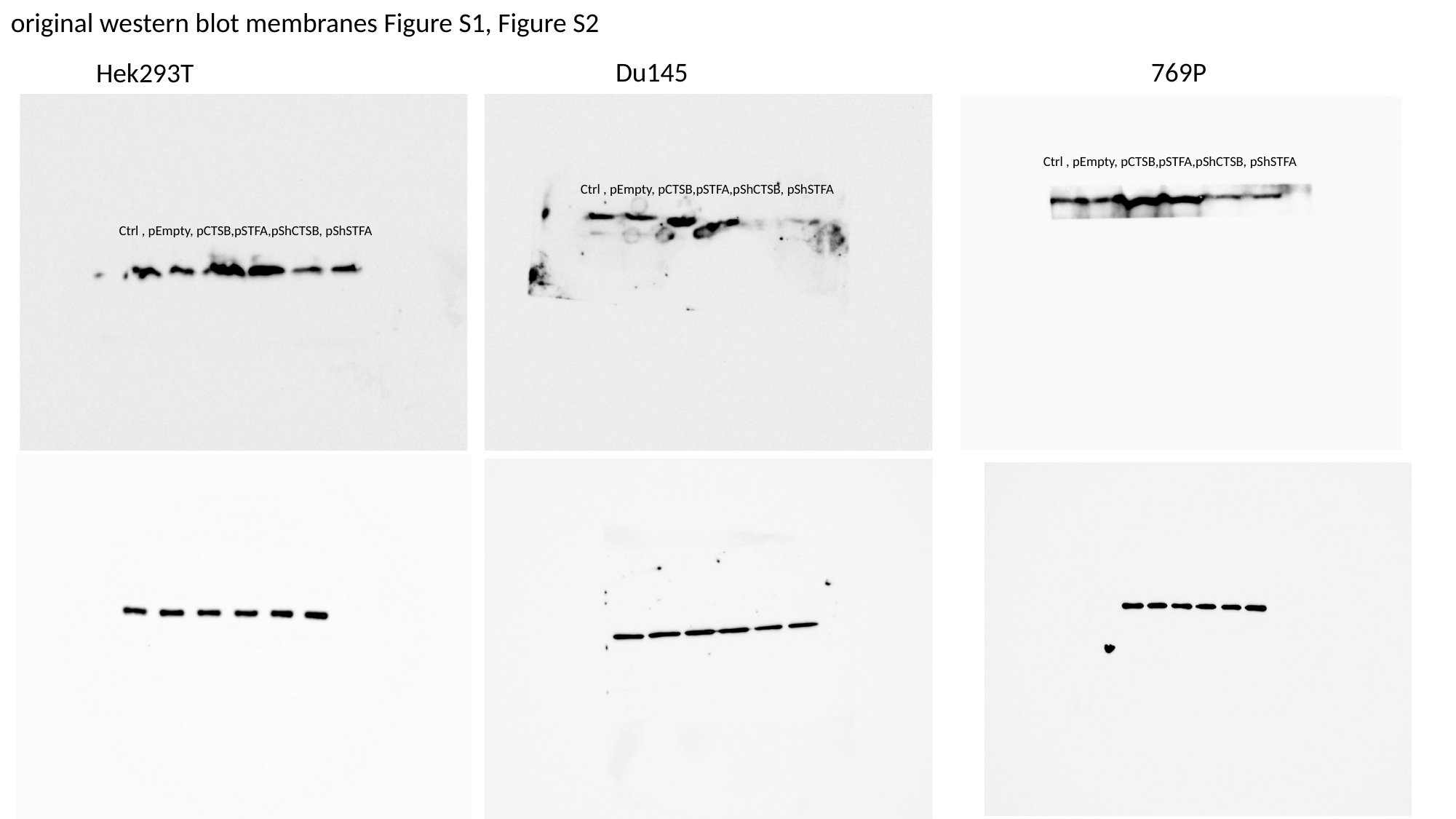

original western blot membranes Figure S1, Figure S2
Du145
769P
Hek293T
Ctrl , pEmpty, pCTSB,pSTFA,pShCTSB, pShSTFA
Ctrl , pEmpty, pCTSB,pSTFA,pShCTSB, pShSTFA
Ctrl , pEmpty, pCTSB,pSTFA,pShCTSB, pShSTFA

## Slide 8
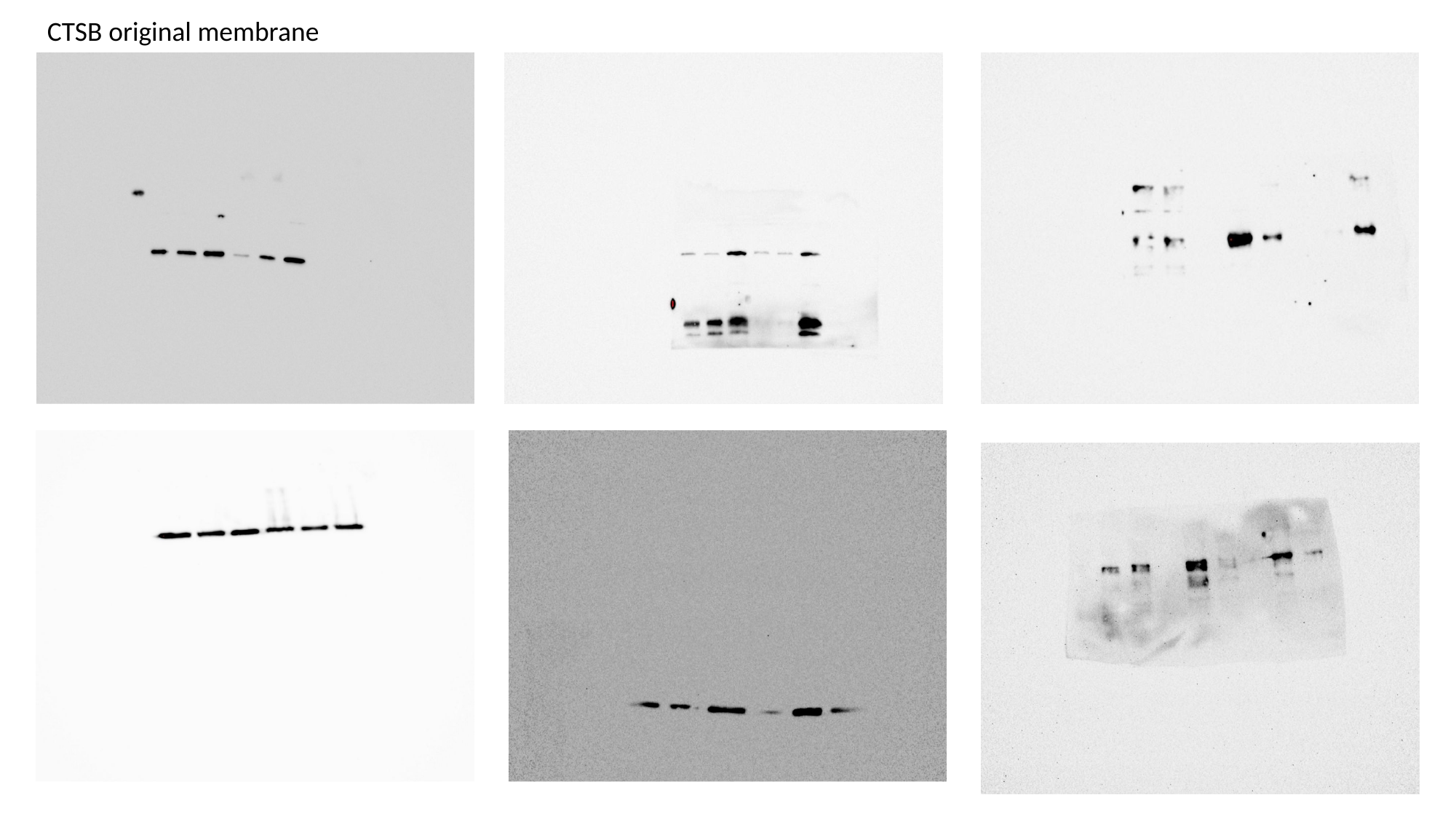

CTSB original membrane

## Slide 9
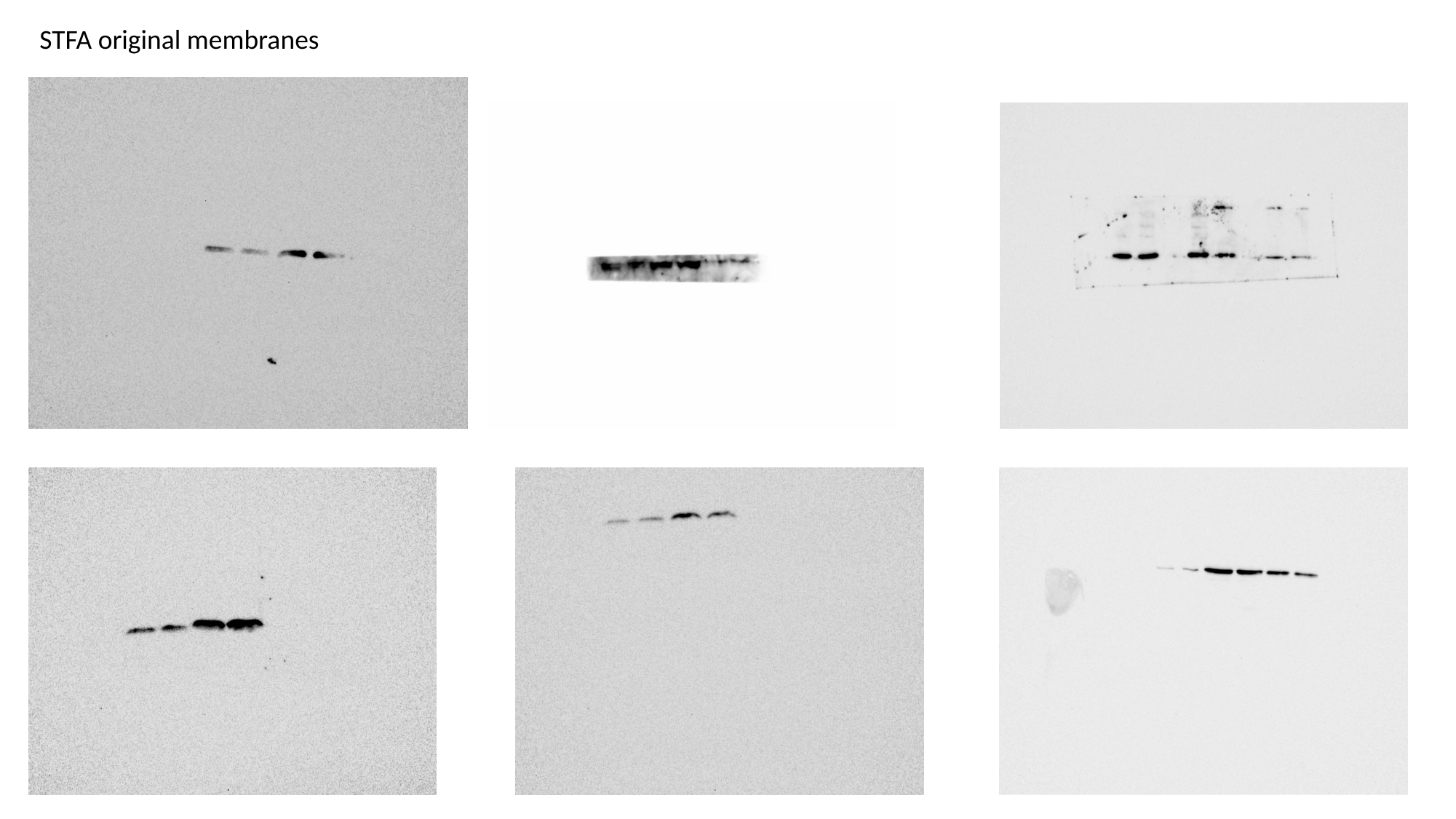

STFA original membranes
